# Unravelling the Silence: A Case Report on the Late Diagnosis of Language-Predominant Frontotemporal Dementia in a Rural Tertiary Hospital

**DOI:** 10.7759/cureus.66183

**Published:** 2024-08-05

**Authors:** Rishitha Kotla, Amol Andhale, Tushar Patil, Sakshi S Dudhe, Devyansh Nimodia

**Affiliations:** 1 Department of Psychiatry, Jawaharlal Nehru Medical College, Datta Meghe Institute of Higher Education and Research, Wardha, IND; 2 Department of Neurology, Jawaharlal Nehru Medical College, Datta Meghe Institute of Higher Education and Research, Wardha, IND; 3 Department of Radiology, Jawaharlal Nehru Medical College, Datta Meghe Institute of Higher Education and Research, Wardha, IND; 4 Department of Radiodiagnosis, Jawaharlal Nehru Medical College, Datta Meghe Institute of Higher Education and Research, Wardha, IND

**Keywords:** memory errors, dementia, behavioral changes, language predominant, frontotemporal dementia

## Abstract

Frontotemporal dementia (FTD) is one of the significant neurological disorders that mostly affects over-60-year-old adults. In essence, FTD, which results from frontal and temporal lobe damages, manifests itself in several ways that include behavioral modifications as well as linguistic loss. These are behavioral variant FTD (bvFTD), primary progressive aphasia (PPA), or various movement disorders with genetic links. FTD takes, on average, three years to be diagnosed since there are no definitive diagnostic tests for this disease. MRI and PET scans use brain imaging techniques to observe damaged parts of the brain. The case study shows a lot of deep-seated language deficits and memory impairments, which ultimately point to the involvement of the temporal lobe. Understanding about FTD and early detection are crucial in enhancing intervention as well as management efforts.

## Introduction

Frontotemporal dementia (FTD) is a form of dementia that targets mainly the frontal and temporal parts of the brain, causing a progressive decline in social behavior, personal traits, speech, and/or muscle movement. FTD differs from other forms of dementia in that it tends to affect younger individuals (usually between 40 and 65) and is characterized by marked changes in social skills as well as emotional functioning. Typical manifestations include indifference, recklessness, compulsion to do certain acts or say certain things, problems with language use, and poor cognitive abilities [1]. FTD itself is characterized by the abnormal aggregation of proteins within the brain [2]. There are no definite means of curing FTD, but early detection and optimal management can help enhance the functional abilities of people affected by the disorder. More studies are still being conducted on what leads to FTD as well as its treatment options [3].

## Case presentation

A 63-year-old married male, a farmer by occupation, was brought to the rural hospital by his relatives, who reported difficulty in speaking, which according to them started three years ago. Pre-morbidly, he was an extrovert and hardworking ex-military serviceman who had good social interactions but gradually started withdrawing himself from family members. He also stopped working on the farm after the onset of memory difficulties like executive deficits (managing finances, multitasking, and taking decisions) and learning deficits (difficulty recalling and repeating and having to be constantly reminded about misplacing and losing items), which were initially observed. As it was during the pandemic period, the relatives had left it unnoticed. It only came to light when his speech and perceptual functions also started declining (difficulty finding words, forgetting names, alternate usage of words). An academically merited student like him making multiple grammatical errors and having minimal social interaction with no interest in activity at home (paying bills or attending events) was concerning enough to take him to a private practitioner, where he was initially diagnosed with Alzheimer's disease. He used to be a very tidy man before the disease, but as the symptoms started progressing, his personal hygiene also declined substantially, causing him to soil the bed and only change clothes or shave when asked by relatives. There was a considerable change in his appetite; initially, he started eating less, but in the past few months, he has had an abnormal pattern of eating at an increased pace. He occasionally presented with abnormal behavior (standing on the bed and an occasional aggressive nature).

The patient had no history of head trauma; however, he was an alcoholic with tobacco use, which he abstained from for eight years. He was also diagnosed incidentally when he went for a routine checkup with hypertension five years ago and reports being on regular medication. There was no significant family history of any similar complaints or other medical and psychiatric conditions. In the mental status examination, he was alert, fully oriented, and cooperative, but his speech pace and quality have decreased to a wide range, where he is coherent but irrelevant with tangentiality present. His spontaneous attention was impaired, and his affect was flat with complex attention (digit span and serial subtraction), abstract and arithmetic, and memory (immediate, registration, and recall) impairments. His cognitive abilities were also seen to have stereotypical and compulsive behaviors. However, thought and perception were deferred as he had difficulty making words. There were a few stereotypical behaviors, like repetitive hand movements, which were not goal-directed, and no insight into his condition.

On a general physical and neurological examination, the patient was seen to have coarse tremors, cogwheel rigidity, and gait abnormality (festinant gait with a short stepping pattern without axial rigidity), which, on further evaluation, was deduced to have Parkinsonian symptoms. There was visual agnosia but no nystagmus, and the Mini-Mental State Examination score was 5/30 (which was initially 8/30) in the neuropsychiatric examination. The Montreal Cognitive Assessment (MOCA) scoring was done in which impairment was observed in visuospatial/executive, naming, memory, language, and delayed abstraction, giving a score of 4/30.

**Figure 1:**
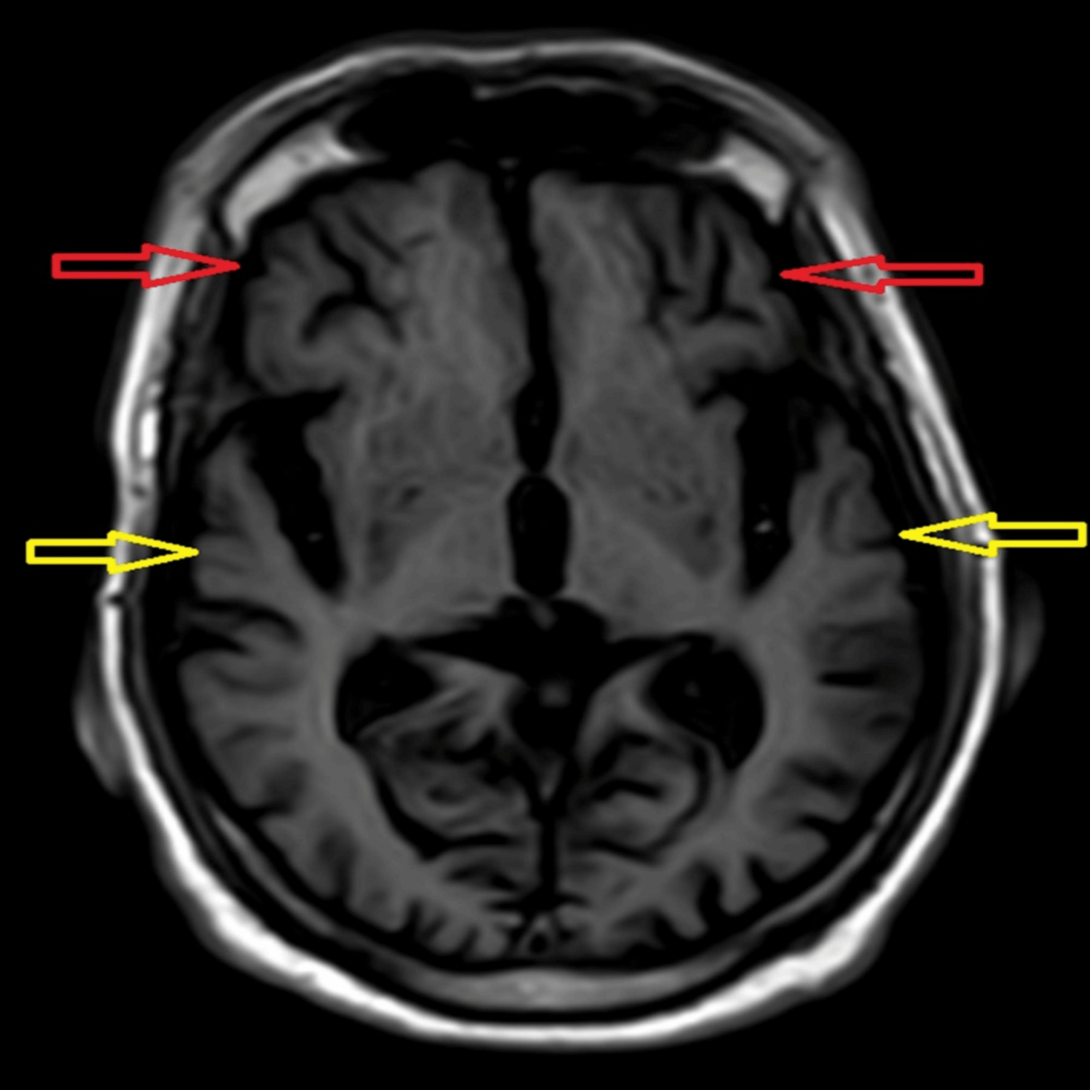
MRI in T1-weighted axial section showing atrophy of the bilateral frontal lobe (red arrows) and temporal lobe (yellow arrows)

**Figure 2:**
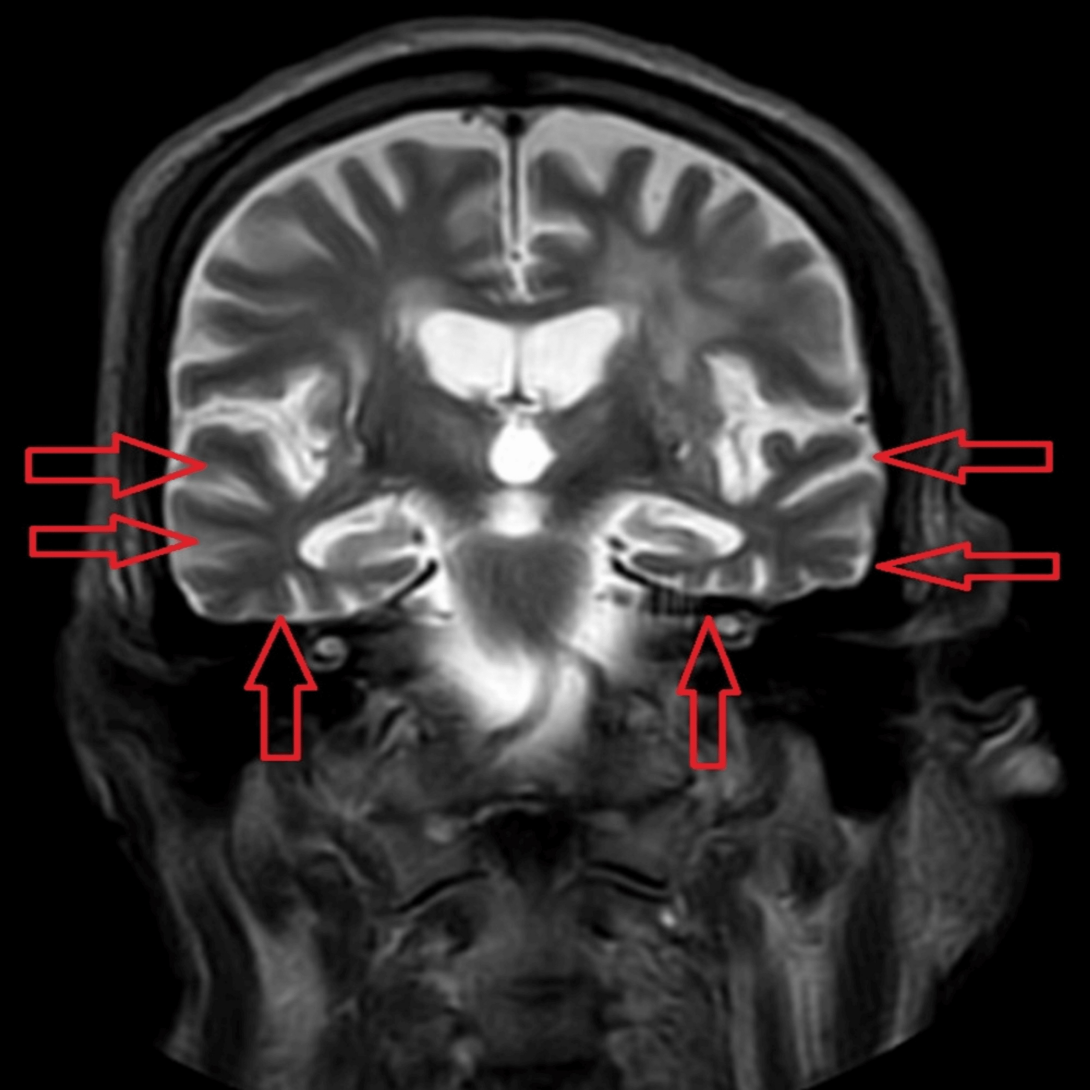
MRI in T2-weighted coronal section showing age-related atrophic changes in the bilateral frontotemporal lobe with thinning of the bilateral superior, middle, and inferior temporal gyri (red arrows)

In laboratory tests, routine investigations were done, and liver, thyroid function, and vitamin D were within normal limits, but the patient had a mild derangement in vitamin B12 values (231) and renal function (creatinine=2.2, sodium=123). Venereal disease research laboratory test (VDRL), hepatitis B surface antigen (HBsAg), anti-hepatitis C virus (anti-HCV), and human immunodeficiency virus (HIV) profiles also came back negative. His chest X-ray, urinalysis, electrocardiogram (ECG), and routine electroencephalogram (EEG) were normal. In the MRI, there was atrophy seen predominantly in the dorsolateral and orbitofrontal areas bilaterally, leading to the diagnosis of language-predominant FTD. Figure 1 and Figure 2 below are the neuroimaging findings of the patient, showing atrophic changes suggestive of FTD. 

A trial of oral Cogtin Plus (*Ginkgo biloba*, piracetam, and vinpocetine) and Donep-M (donepezil and memantine combination) was initially tried by a private practitioner two years ago for four months to combat the memory impairment with minimal improvement. Tab. Syndopa Plus (levodopa and carbidopa combination) was given for Parkinsonian symptoms. With conservative treatment and the above medication, his self-care and communication partially increased. The patient, hence, was discharged under hemodynamically stable conditions and was psychoeducated about medication compliance until the next follow-up.

## Discussion

According to a 2023 study, the prevalence of dementia in India is 7.4% for adults aged 60 or older, meaning 8.8 million are affected by it [4]. Also known as Pick's disease, FTD is a variant of dementia that is a degenerative brain disease causing changes in behavior and language. It is a progressive neurological syndrome with a diverse clinical presentation, along with neuropsychiatric manifestations and linguistic deficits, mostly affecting the anterior temporal and frontal lobes [5]. It is considered the third most common dementia for individuals aged 65 years and older, having three kinds of clinical subtypes: behavioral variant FTD (bvFTD), primary progressive aphasia (PPA) (progressive non-fluent aphasia and semantic dementia), and movement disorder [6]. The causative factor is unknown; however, researchers have linked certain subtypes to genetic predisposition as an underlying cause [7]. Some patients have been reported to have abnormal intraneuronal inclusion bodies caused by protein accumulation (tau protein (or) tangles) called Pick's bodies [8].

According to the Association for Frontotemporal Degeneration (AFTD), it takes an average of three years to get a definitive diagnosis of FTD. In bvFTD, as the frontal lobe is affected, it causes personality and behavioral changes with common signs such as inappropriate social behavior, loss of apathy, lack of judgment, loss of inhibition, repetitive compulsive behavior, and drastic changes in personal hygiene [9]. But in PPA, there is difficulty in speaking, writing, and/or understanding language [10]. Disorders like amyotrophic lateral sclerosis, corticobasal syndrome, and progressive supranuclear palsy are also associated with FTD [11]. Hence, investigations like MRI and PET scans play a vital role in diagnosing the specific region affected and further facilitating the early management of the underlying causes [12].

In our case, there have been more language deficits, along with memory impairment, compared to the behavioral variant. The temporal lobe is known for playing a role in managing emotions, processing information and memories, and understanding language, whereas the frontal lobe is mainly responsible for expressing language and higher executive functions such as planning, organizing, and self-monitoring [13].

## Conclusions

Understanding the significant prevalence and impact of FTD, especially with subtypes like bvFTD and PPA, underscores the need for heightened awareness and comprehensive diagnostic approaches. Diagnostic tools like MRI and PET scans are essential in identifying affected brain regions, aiding in timely diagnosis and treatment.
